# MiTra: A Drone-Based Trajectory Data for an All-Traffic-State Inclusive Freeway with Ramps

**DOI:** 10.1038/s41597-025-05472-0

**Published:** 2025-07-09

**Authors:** Ankit Anil Chaudhari, Martin Treiber, Ostap Okhrin

**Affiliations:** 1 Faculty of Transport and Traffic Sciences, Technische Universität Dresden, Dresden, Germany; 2 Center for Scalable Data Analytics and Artificial Intelligence (ScaDS.AI), Dresden/Leipzig, Germany

**Keywords:** Computer science, Civil engineering

## Abstract

Traffic flow modeling is essential for transportation engineering and urban planning, particularly in complex scenarios such as lane-changing and movements at ramps. However, obtaining high-quality trajectory data remains challenging, especially in urban environments where traditional methods like stationary cameras fall short. Existing drone-based datasets often lack full coverage of traffic states and critical merging and diverging behaviors at ramps. This study presents MiTra (Milan Trajectories), a high-resolution traffic trajectory dataset collected using six drones over a 900 m section of the A50 urban freeway in Milan, Italy. Spanning all traffic states from free flow to congestion, it captures detailed vehicle behavior at on-ramps and off-ramps through nine flight campaigns for 135 min. It includes 124 641 vehicle trajectories, averaging 650 m in length after stitching, with detailed positions, speeds, and accelerations. Nearly half of the vehicles executed lane changes. The dataset provides stitched trajectory data, raw drone videos, and tracking logs. Comprehensive quality checks, including vehicle detection and video stitching validation, ensure its reliability for traffic modeling, autonomous driving research, and computer vision applications.

## Background & Summary

Understanding traffic dynamics is crucial for developing effective urban planning strategies, traffic management systems, safety analysis, emission prediction, and autonomous vehicle technologies. Traffic data can generally be categorized into two main types based on the data acquisition method: stationary data and floating-car data. Stationary data is collected from fixed observation points, either at a specific cross-section (e.g., stationary loop detectors or cameras, or Bluetooth, wifi, or RFID sensors), at a fixed time (snapshots), or over a spacetime region (e.g., trajectory data). This category includes both macroscopic data, aggregated over time intervals (e.g., typically one minute, providing flow, density, and average speed), and microscopic data, which captures individual vehicle movements. Floating-car data, on the other hand, provides vehicle-specific information, typically obtained from in-vehicle sensors, GPS systems, or probe vehicles^1^. This type of data, often referred to as “perception data” in the context of autonomous vehicle development, is primarily used to understand vehicle-level behavior and train perception algorithms for tasks like object detection and scene interpretation. While highly detailed, floating-car data does not offer a complete picture of traffic flow across multiple vehicles and regions simultaneously. Each data type offers unique insights but has limitations in its application and resolution. Loop detector data, a common type of macroscopic data, provides aggregated information such as traffic flow and average speed over specific road segments from which the density can be estimated. This data is valuable for monitoring overall traffic conditions and supports decision-making at the network level, such as optimizing traffic signal timings and managing freeway ramp metering^2^. While macroscopic data is essential for real-time traffic management and congestion detection, it lacks the granularity to understand individual vehicle behaviors or detailed traffic dynamics. Consequently, it is less suitable for safety analysis or studying complex behaviors such as lane-changing and vehicle interactions at ramps. In contrast, microscopic trajectory data captures the detailed movement of individual vehicles over time, providing information on vehicle positions, speeds, accelerations, and lane changes^3^. This type of data is indispensable for modeling and simulating various aspects of traffic flow, including car-following behavior, lane-changing maneuvers, and vehicle interactions^4^. Trajectory data is particularly useful in developing advanced driver-assistance systems (ADAS) and autonomous vehicle algorithms, validating traffic flow models, conducting safety analyses, and understanding the impact of different traffic management strategies^3,5^. Unlike macroscopic data, microscopic data can reveal how individual drivers behave under varying traffic states—from free flow to congestion—and how they interact with infrastructure elements like on-ramps and off-ramps. Perception-based datasets^6–9^ focus on sensor data (e.g., LiDAR, cameras, radar) from a vehicle’s perspective. These datasets are primarily used to train and test autonomous vehicles’ perception algorithms for object detection, tracking, and scene understanding. While crucial for developing the sensory capabilities of autonomous vehicles, perception data offers a limited view of traffic dynamics. It provides information only from the perspective of a single vehicle, missing the broader interactions between multiple vehicles, especially in complex scenarios like merging or lane-changing at ramps. This is where microscopic trajectory data fills a critical gap by providing a holistic view of traffic interactions across multiple vehicles.

Naturalistic traffic data, capturing real-world traffic behaviors, is essential for developing robust traffic flow models and autonomous vehicle systems. However, naturalistic data cannot be created in simulations or test tracks, as these environments lack the complexity of real-world driving scenarios. Traditional data collection methods using stationary cameras, helicopters, or loop detectors have significant limitations: Stationary cameras mounted on buildings or bridges often suffer from restricted views and limited coverage, particularly in urban areas with complex geometries^10,11^. Helicopters provide a wider view but result in unstable footage and are unsuitable for long recording durations due to high costs and operational constraints. Loop detectors only capture aggregate data at specific points, lacking the resolution to track individual vehicle movements or interactions, especially during lane changes or merging at ramps. These limitations make it challenging to capture the detailed vehicle behaviors necessary to develop accurate traffic models and study complex traffic scenarios.

Drone technology offers several advantages over traditional data collection methods. Drones provide a top-down perspective and high-resolution footage, which allows for capturing detailed vehicle trajectories over large sections of roadway^12–14^. Unlike stationary cameras or helicopters, drones are highly flexible and can operate in areas where fixed installations are impractical. They can fly over complex road geometries, including ramps and intersections, to record vehicle movements with minimal disruption to traffic flow. Using multiple drones makes it possible to cover larger sections of the road while maintaining the spatial resolution needed for trajectory analysis. This is particularly important for capturing behaviors at on-ramps, off-ramps, and weaving sections, where frequent lane changes and merging occur. Drones allow for the collection of continuous trajectory data across all traffic states, from free-flow to congestion, enabling more comprehensive studies of traffic dynamics than those based on loop detectors or stationary cameras.

### Comparison with previous datasets

While previous research utilizing drone-based data collection has shown promise, existing datasets^15–27^ highlight significant gaps in terms of the ability to capture diverse traffic states and various infrastructure elements. Table 1 compares various trajectory datasets covering long road sections with merging and diverging scenarios.

**Table 1:** Comparison of existing trajectory datasets from lane-based traffic.

Dataset	Road Section Condition	Country	Vehicle Types	Motorised Vehicle Trajectories	Record Distance/Area	Record Duration	Frame Rate	Collection Device
**NGSIM (2008)**^28^	Freeway/Merging-diverging ramps/Intersections	USA	Cars and Heavy Vehicles	9 206	488 m-640 m	2.5 h at 4 locations	10 Hz	Multiple Stationary Cameras
**HighD (2018)**^15^	Freeway	Germany	Cars and Heavy Vehicles	110 000	420 m	16 h at 6 locations	25 Hz	Single Drone
**Interaction (2019)**^16^	Freeway/Roundabout/Intersections	USA, Germany, Bulgaria, China	Multi Types	10 933 (merging and lane change)	Various	2.22 h at 2 locations (Germany and China)	10 Hz	Single Drone
**pNeuma (2020)**^17^	Urban Network	Greece	Multi Types	500 000	1.3 km^2^	2.5 h for 5 days	25 Hz	Multiple Drones
**ZenData (2020)**^39^	Urban Highway	Japan	Cars and Heavy Vehicles	18 000	2 000 m	5 h	10 Hz	Multiple Stationary Cameras
**Automatum (2021)**^18^	Freeway	Germany	Cars and Heavy Vehicles	595 444	—	30 h at 12 locations	—	Single Drone
**HighSIM (2021)**^40^	Freeway	USA	Cars and Heavy Vehicles	2 182	2 438 m	2 h	30 Hz	Helicopter with 3 cameras
**Magic (2022)**^19^	Freeway	China	Multi Types	—	2 000 m (non-stitched)	3 h	25 Hz	Multiple Drones
**exid (2022)**^22^	Freeway/Merging-diverging ramp	Germany	Multi Types	69 172	—	16 h at 7 locations	25 Hz	Single Drone
**CitySim (2022)**^23^	Freeway/Merging-diverging ramps/Weaving sections	USA	Multi Types	—	Various	5 h at 4 locations	30 Hz	One or Two Drones
**City Scale (2023)**^5^	Urban Network	China	Multi Types	3 439 684	5 districts and Whole City	12-24 h for 4 days	—	Multiple Stationary Cameras
**I-24 (2023)**^41^	Freeway	USA	Multi Types	600 000	6 750 m (non-stitched)	47 h	25 Hz	Multiple Stationary Cameras
**TUMDOT-MUC (2024)**^24^	Urban road	Germany	Multi types	10 531	700 m	7 h	25 Hz	Multiple Drones
**MiTra (this contribution)**^29^	Freeway/Merging-diverging ramps	Italy	Multi types	124 641	900 m	2.25 h	30 Hz	Multiple Drones

Many of these datasets lack the comprehensive spatial and temporal coverage required for a detailed driving behavior analysis across various traffic conditions. NGSIM^28^ often lacks resolution and has numerous recording artifacts^10,11^. For instance, the pNeuma dataset^17^, collected in Athens, Greece, offers valuable insights into city traffic but suffers from interruptions due to non-recorded traffic signals at intersections, limiting the continuity of vehicle trajectories. Similarly, the HighD dataset^15^ focused on freeway, and the ExiD dataset^22^, which records vehicle entries and exit, were both recorded using a single drone and primarily captures free-flowing traffic conditions over relatively short road sections (around 420 m), thus offering limited insights into driver behavior in congested traffic scenarios as well as the whole dynamics of merging and diverging traffic at ramps; as a result, these datasets fall short in capturing the full range of driver behaviors, especially in more complex traffic situations like congestion. The MAGIC dataset^19^ was recorded in Shanghai using six drones, which provided a more extensive recording area. However, the data from each drone is available separately rather than being stitched together into a single dataset, which prevents the analysis of longer, continuous vehicle trajectories that span multiple traffic stages. This data segmentation limits the ability to conduct comprehensive analyses of how vehicles transition between different traffic states, such as free-flow to congestion, over longer distances. Similarly, the Interaction dataset^16^, which focuses on freeways, roundabouts, and intersections in multiple countries, includes only around 10 933 motorized trajectories for highways with merging and lane-changing behavior. This limited number of trajectories restricts the dataset’s capture of various traffic conditions. Other datasets like inD^20^ and rounD^21^ focus exclusively on intersections and roundabouts, respectively, and are thus not well-suited for highway analysis or studying merging and diverging behaviors. The Automatum dataset^18^, while extensive in terms of the number of trajectories recorded (over half a million), uses only a single drone, making the study section too small to understand traffic dynamics fully. More recently, the TUMDOT-MUC dataset^24^ was collected with six drones in Munich, Germany. Although it captures traffic with spatial and temporal connectivity, the road section contains an intersection and traffic signals, limiting its applicability for studying freeway dynamics or uninterrupted vehicle trajectories over long sections. Similarly, the CityScale dataset^5^ focuses on city intersections across two cities in China, relying on 441–1838 stationary cameras to collect data, which, while extensive, is aggregated.

However, despite the value these datasets bring to traffic analysis, they all face limitations regarding coverage, continuity, and the ability to capture complex traffic scenarios. In particular, there remains a significant gap in datasets that can provide:

- comprehensive spatial and temporal continuity, allowing for the study of vehicle interactions across a larger road network;
- detailed analyses of merging, diverging, and lane-changing behaviors at on-ramps and off-ramps are critical for understanding bottlenecks and congestion formation;
- continuous and seamless captures of the full spectrum of traffic states, from free flow to congestion.

These points were the primary motivation for our data collection campaign. By leveraging drone technology, we overcame many challenges associated with traditional data collection methods. This approach allowed us to capture detailed traffic data across all traffic states, including critical behaviors at on-ramps and off-ramps.

In this study, we focus on a section of the highway in Milan, Italy, one of Europe’s largest and most densely populated cities. This location provides an ideal setting to study traffic behavior in a real-world urban environment. Our ***MiTra*** **(Milan Trajectories)** dataset^29^, collected over a 900 m section of the A50 urban freeway in Milan, Italy, spans nine flight campaigns using six drones, covering various traffic conditions from free-flowing to heavily congested traffic. The dataset includes 124 641 vehicle trajectories, with detailed information on vehicle positions, speeds, and accelerations. The data was meticulously validated through processes such as vehicle detection accuracy and video stitching, ensuring its reliability and usefulness for traffic analysis.

We contributed to the research on transportation by creating a validated, high-resolution traffic trajectory dataset that captures various traffic conditions and behaviors. This dataset provides a valuable resource for researchers and practitioners in traffic flow modeling, autonomous vehicle development, and traffic management. Potential applications of our dataset include enhancing traffic simulation models, studying lane-changing behavior, and analyzing traffic transitions at critical points such as on-ramps and off-ramps. Moreover, most datasets above do not provide raw videos or tracking files. These resources are essential for further research, especially in computer vision. Our dataset also provides raw video recordings of the drone footage, which will benefit researchers and developers working on vehicle detection and trajectory identification. Computer vision scientists can use this raw video data to create or train new models for detecting vehicles in real-world scenarios, improving accuracy and robustness in complex traffic conditions. In addition to the raw videos, we also offer tracking files generated during the trajectory extraction process. These tracking files are vital for researchers who want to cross-check the video data with the extracted vehicle trajectories, ensuring accuracy and consistency. This combination of raw video data and tracking logs will support a broader range of research, from traffic analysis to developing advanced detection and tracking algorithms.

Looking ahead, our dataset opens up new avenues for research. Future studies could build on this work by developing more sophisticated traffic models, improving the accuracy of autonomous driving algorithms, or devising new strategies for traffic management that can adapt to dynamic traffic conditions. By providing a robust data foundation, our work contributes to the ongoing efforts to understand and optimize traffic flow in increasingly complex and congested urban environments.

## Methods

### Traffic Recording Campaign Design and Execution

The objective of this campaign was to capture comprehensive traffic trajectory data on the A50 freeway in the Rozzano District, Milan, Italy, especially with the sections involving merging, diverging, and lane-changing behaviors. The dataset aimed to cover the full spectrum of traffic states, from free-flowing conditions to complete congestion, capturing the stop-and-go waves crucial for studying different traffic phenomena, particularly in modeling and simulation.

#### Road Section Selection

**Fig. 1:**
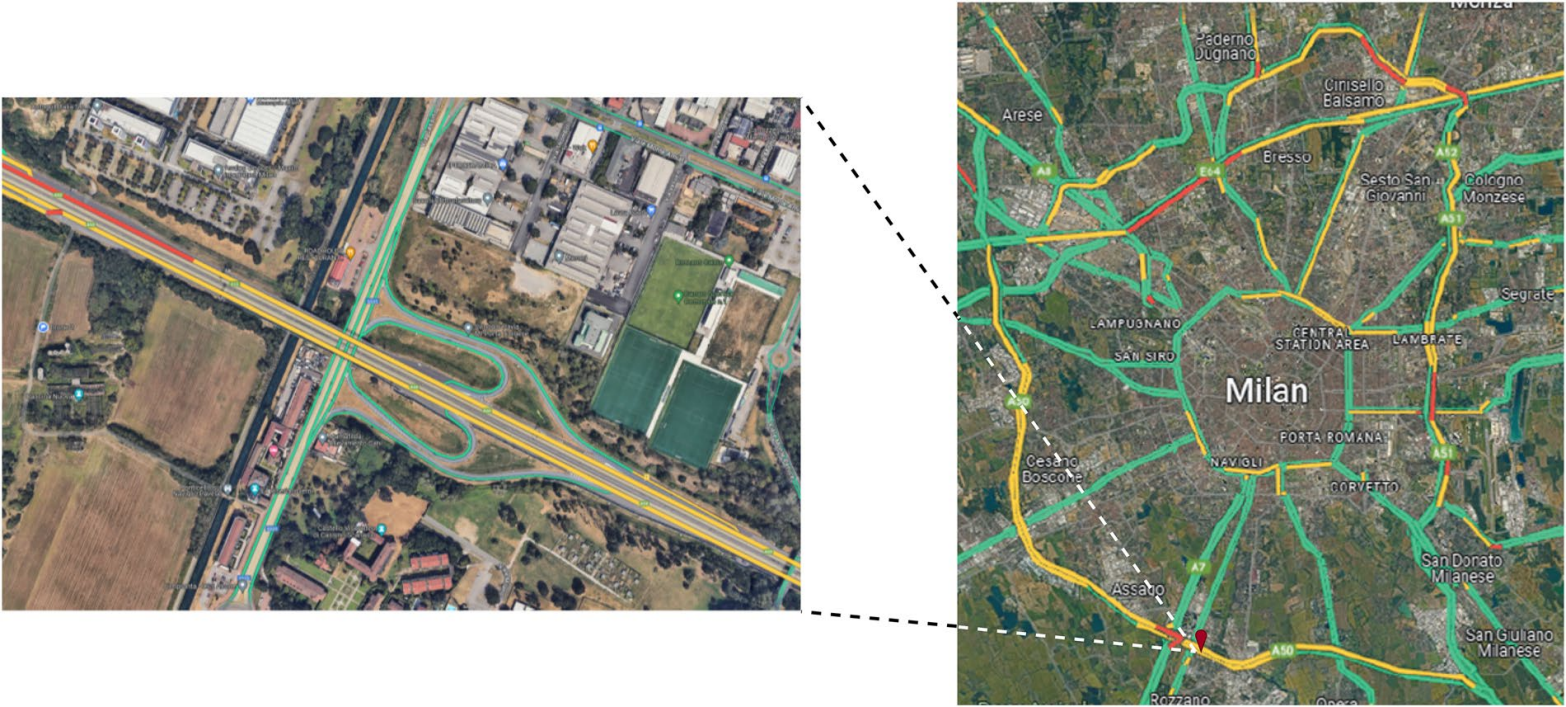
Study Section (Google Maps).

The first critical design task was selecting a road section meeting several key criteria: multiple lanes, the presence of on-ramps and off-ramps to study merging and diverging behavior, and a continuous stretch free from disruptions such as bus stops or traffic signals. The chosen section needed to be long enough to capture continuous vehicle trajectories, as previous research, such as Wang *et al*.^30^, indicated that continuous trajectories of more than 30 s are essential for real-time safety analysis, as some variables in 0–1 min intervals before the crash have a positive impact. The A50 freeway, as shown in Fig. 1, a 900 m stretch with four ramps (two on-ramps and two off-ramps) and six lanes plus additional lanes for ramp connections, was selected as it satisfied these requirements and adhered to the drone flying regulations set by the European Union Aviation Safety Agency (EASA)^31^, Italian Civil Aviation Authority (ENAC)^32^, and ENAV^33^.

#### Timing of Data Collection

Selecting the appropriate day and time was another crucial factor, as the experiment required capturing a range of traffic states, from free-flow to congestion. Using the “Typical Traffic” feature of Google Maps, it was determined that Thursday evening peak hours, starting from 17:30, would likely produce the desired congested traffic conditions. Therefore, the recording was scheduled from 15:15 onwards to capture the transition from free-flow to congested conditions. The data collection was initially planned for April 13, 2023, but was postponed due to rain forecasted on both April 13 and April 20. The recording was rescheduled to April 27, 2023, when favorable weather conditions were expected.

#### Weather Considerations

The weather on April 27, 2023, was monitored closely to ensure optimal conditions for the experiment. The temperature was 18^°^ C, and the wind speed was 13 km/h. These conditions were considered ideal, with the mild temperature ensuring that drone operations would not be affected by overheating and the moderate wind speed providing sufficient stability for the drones during flight, ensuring high-quality video recordings.

#### Drone Setup and Flight Planning

A critical preparatory step was determining the appropriate number of UAVs required to cover the desired road section and identifying the exact hovering locations for each UAV. A test flight was conducted to ensure that the UAVs were positioned correctly, providing adequate spatial overlap to allow for accurate vehicle re-identification during the video stitching process. The trajectory extraction team reviewed the test flights to confirm that the UAVs were positioned at the correct locations and angles for optimal trajectory extraction.

#### Execution of Data Collection

**Fig. 2:**
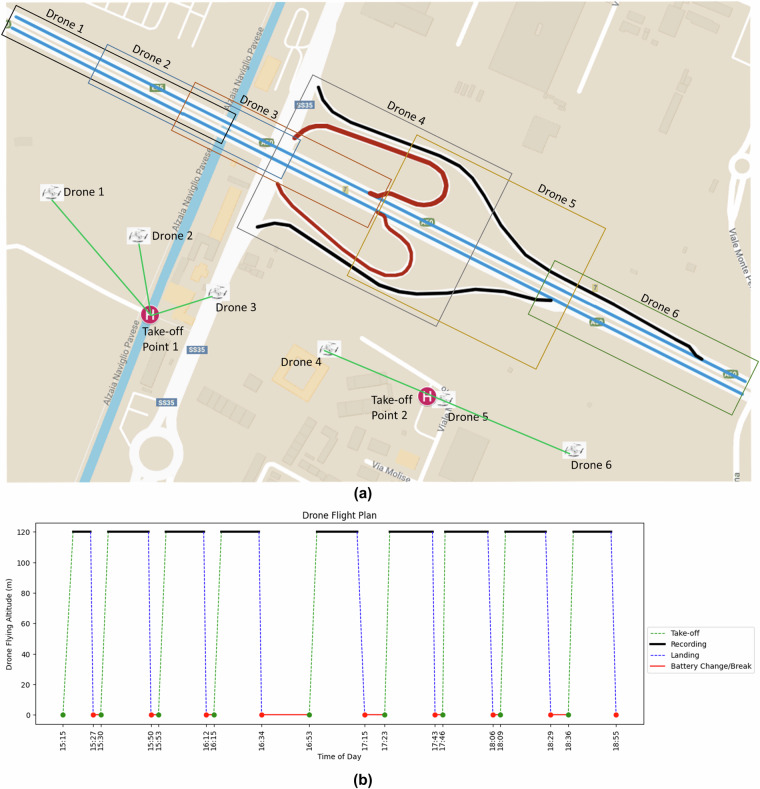
Data recording campaign design. (**a**) Drone hovering points and take-off/landing location; (**b**) Drone Flight Plan.

A professional UAV flight team was employed to ensure the experiment’s success. The team consisted of seven pilots and one central coordinator, responsible for obtaining necessary permissions and ensuring a smooth operation. The day before the experiment, detailed instructions were provided to the flight team to ensure organized and coordinated execution. The experiment was executed using six DJI Mini 2 drones, recording at 30 frames per second with a 4K (4096 × 2160) resolution. Based on the test flight findings, the drones were deployed at a height of 120 m, in compliance with EASA regulations. Drones 1, 2, and 3 took off and landed from Location 1, while Drones 4, 5, and 6 operated from Location 2, as shown in Fig. 2(a), along with the road section covered under each drone. The hovering locations were strategically chosen to ensure sufficient overlap between adjacent drones’ recordings, which is crucial for stitching the videos together during data processing.

#### Battery Management and Data Continuity

**Fig. 3:**
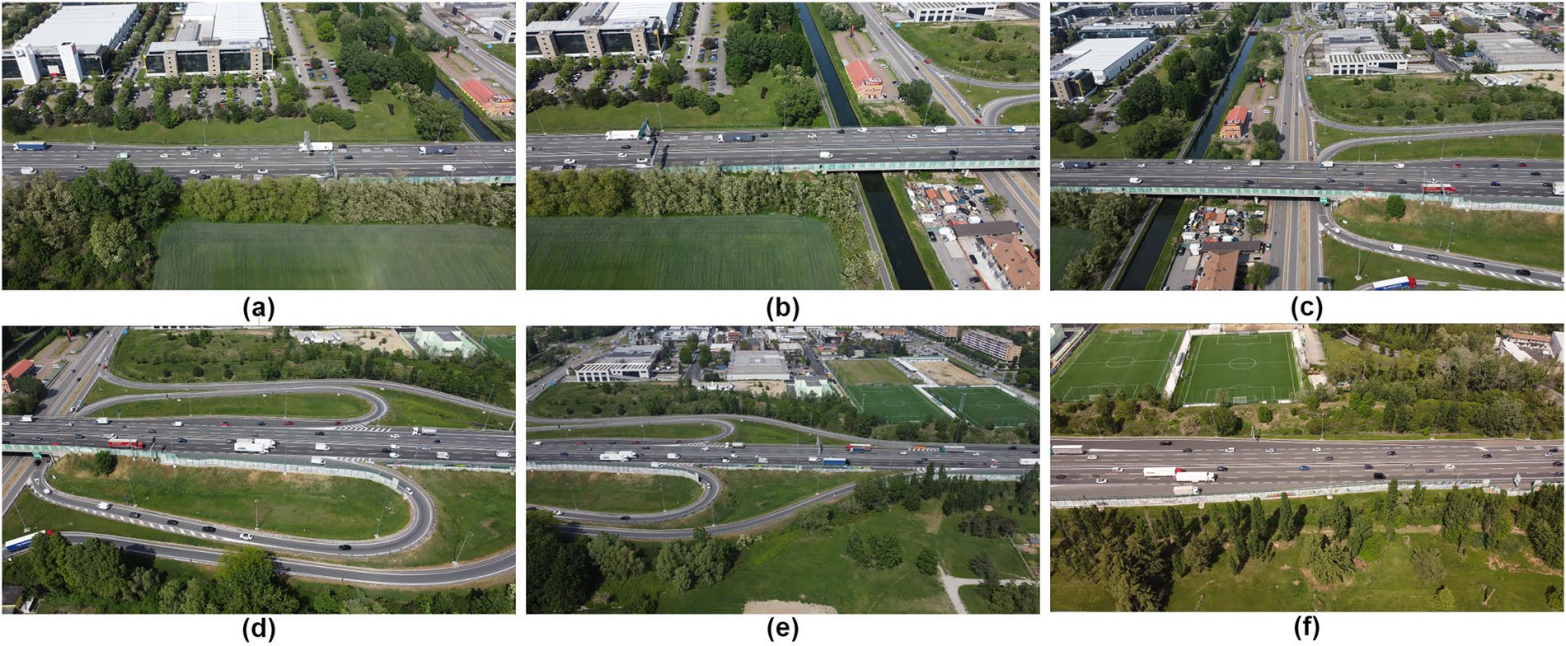
Road sections captured under each drone.(**a**) Drone 1; (**b**) Drone 2; (**c**) Drone 3; (**d**) Drone 4; (**e**) Drone 5; (**f**) Drone 6.

One of the primary challenges was the limited flight time due to their battery capacity. Regarding the temporal protocol, two options were considered: the first involved swapping drones mid-flight to ensure uninterrupted recording by temporal stitching, which would have required twice the number of skilled pilots and complex coordination, thus doubling costs. The second option, which was ultimately chosen, involved allowing the drones to fly until their batteries were low, then landing, replacing the batteries, and resuming recording. Although this approach did not result in temporally continuous data, it was carefully planned to ensure the coverage of all traffic states. Figure 3 illustrates the road sections covered by each of the six drones.

#### Synchronization During Recording

During the experiment, synchronization was critical. All drones took off simultaneously from their respective locations and hovered at their designated points. Once all drones were positioned, the central coordinator signaled the pilots to start recording simultaneously. The drones’ internal sensors, including the gimbal, maintained stability during recording. When a drone battery was low, the pilot notified the central coordinator, who then instructed all pilots to stop recording simultaneously. The drones were then landed, batteries were replaced, and the process was repeated nine times. As shown in Fig. 2(b) in detail, the first flight began at 15:15, and the final flight landed at 18:55, resulting in nine flights with an average duration of 15 min (except the first flight), resulting in 135 min of net recorded data over a span of 3.5 h. The six drones recorded 13.5 h of footage across nine flights.

### Data Extraction Process

Following the successful recording of the drone footage, the raw video files, captured in 4K resolution at 30 frames per second, were immediately transferred to secure storage and backed up to prevent any data loss. The next critical step was to extract traffic trajectory data from these raw videos. This complex task was outsourced to the DataFromSky^34^ company, which is recognized for its expertise in aerial video traffic analysis. Their robust vehicle detection algorithms and high accuracy in tracking vehicles across multiple frames made them ideal for handling the complexities of our dataset, including scenarios with occlusions and stitching of videos from multiple drones.

#### Vehicle Detection and Tracking

The detection process utilized by DataFromSky was based on advanced computer vision algorithms. These networks were meticulously trained on a diverse dataset comprising both real and synthetic images curated by the DataFromSky team. The detection process was primarily automated; however, manual adjustments were made in areas with heavy traffic or occlusions to refine detection accuracy. Detected vehicles were then fed into the Multiple Hypothesis Tracking (MHT)^35^ system, which employed kinematic models and visual similarity algorithms to maintain continuous and precise vehicle trajectories. The MHT tracker also integrated a prediction model, allowing it to filter data and maintain accuracy even in challenging conditions, such as when vehicle images were temporarily occluded or overlapping.

To ensure the integrity and accuracy of the data, the DataFromSky team conducted thorough manual cross-checking by reviewing random samples of the trajectory data and found no identification or tracking errors. Furthermore, they cross-referenced the output with known reference points in the video to ensure spatial accuracy. Any anomalies detected during this process were corrected through manual adjustments.

#### Video Stitching and Trajectory Extraction

Given that the videos were recorded from multiple drones positioned at different locations, the process of video stitching was crucial for creating a coherent, aggregated dataset. One of the primary challenges in stitching these videos was the potential inaccuracy in object localization due to differing viewing angles. Although the effect is less pronounced in top-down views, such perspectives were not possible in our case at some locations due to legislative restrictions. Consequently, sudden shifts in the combined video space, exacerbated by noise, were common issues.

The video stitching was performed at five transitions for 124 641 trajectories within the Universal Transverse Mercator (UTM) coordinate system to address these challenges. Each individual video underwent a geo-registration step, where specific points in the video with known UTM coordinates were used to calculate the transformation matrix. This transformation allowed for recalculating all trajectories and image data into a consistent, absolute coordinate system. The UTM system served as the new aggregation space, where further post-processing of trajectories occurred, including aligning them between overlapping sections and connecting them.

**Fig. 4:**
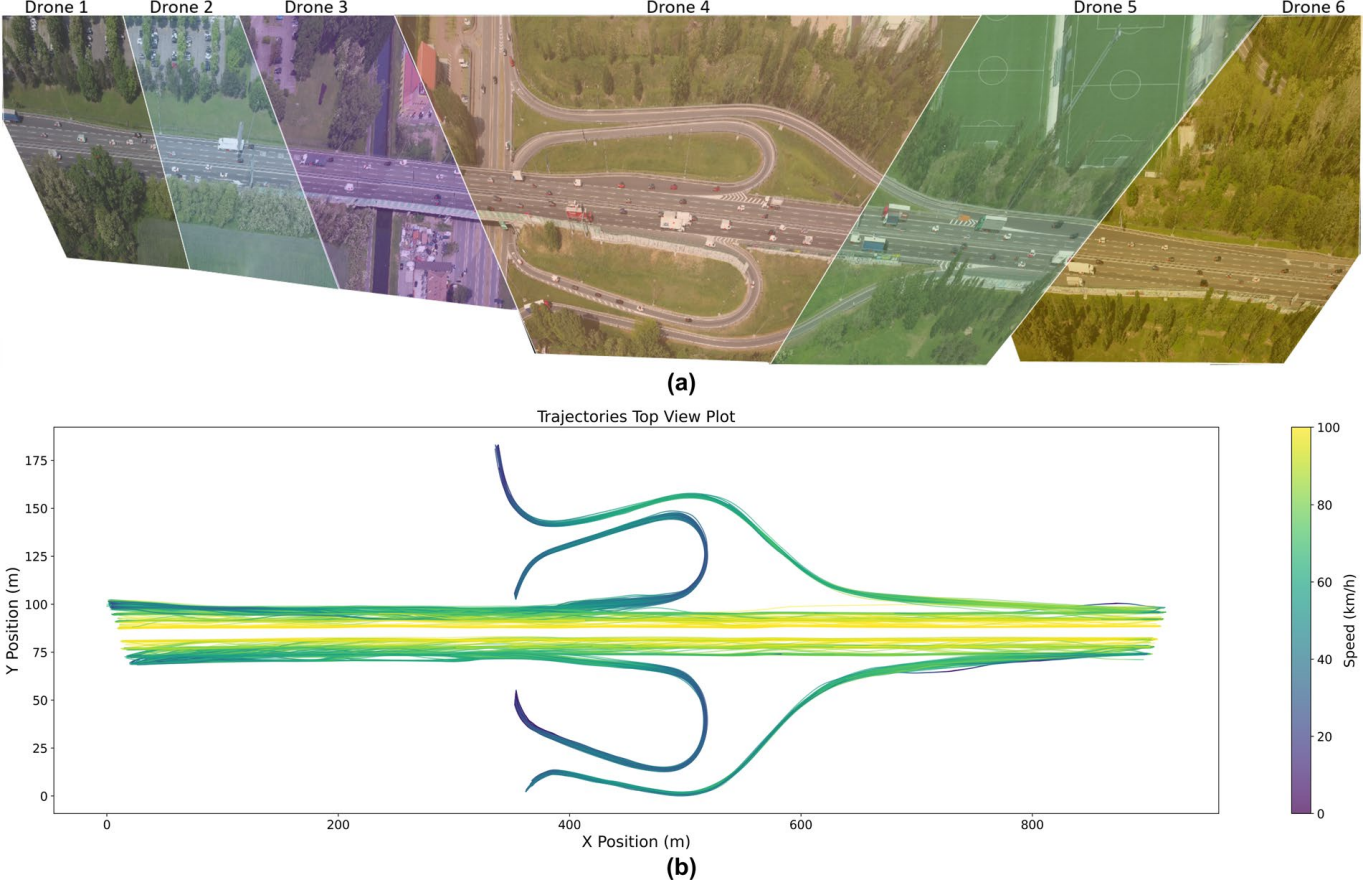
Video Stitching and Extracted Trajectories. (**a**) Study section coverage by stitching six drones videos (Image is made by DataFromSky); (**b**) Extracted Trajectory Data.

Figure 4(a) shows the study section covered under six drones by maintaining proper overlap and stitching. Details about overlapping and stitching validation are provided in the Technical Validation Section. Figure 4(b) shows the extracted trajectories of the whole section; a detailed view of the trajectories can be seen in Fig. 7(h).

#### Trajectory Smoothing and Refinement

Post-processing within the UTM space involved refining the trajectory data to address any inaccuracies caused by differing viewing angles and noise. This was accomplished by using guide trajectories aligned with the traffic lanes, which helped to smooth the final data and significantly reduce the impact of any sudden shifts in object localization. The telemetry data, including vehicle positions, speeds, and accelerations, was initially filtered using a Kalman filter^36^ to reduce noise. In the second phase, trajectory refinement was further enhanced using the guide trajectories, ensuring that the final data closely adhered to the expected vehicle behavior within each lane. The integrity and precision of the extracted data were carefully verified.

#### Privacy and Compliance

The data extraction process was designed to protect privacy. The top-down view of the drones ensured that no identifiable information, such as license plates or faces, was captured. The extracted data focused solely on vehicle trajectories, with no personal data included. All data collection and processing activities complied with European drone regulations^31–33^.

### Descriptive Aspects

From the recorded videos, we successfully extracted 124 641 vehicle trajectories using single drone videos, which encompass 54 datasets corresponding to the nine flight campaigns of six drones and 24 161 trajectories through the stitched footage of all six drones, as shown in Fig. 4(b). The total distance covered by trajectories is more than 20 000 km. We distinguish five categories: Cars (73%), Medium Vehicles (13.4%), Heavy Vehicles (11.3%), Motorcycles (2.1%), and Buses (0.2%). Among the total vehicles, 76.9% moved straight on the highway, while 13% merged and 10.1% diverged from the highway via on-ramps and off-ramps, respectively, in both directions of traffic. For the lane changing, 51.9% of the vehicles within the dataset executed lane changes. Among these, 24.8% underwent a single lane change, while 27.1% changed lanes multiple times. Upon further examination, we observed variations in lane-changing behavior across different vehicle types. Notably, 52.8% of cars, 49.3% of medium vehicles, and 42.3% of heavy vehicles engaged in lane changes, whereas a higher proportion, 85.7%, of motorcycles exhibited this behavior.

## Data Records

The MiTra dataset^29^ is uploaded on the “Open Access Repository and Archive for Research Data of Saxon Universities (*OPARA*)” repository, and it is accessible at 10.25532/OPARA-881. It includes original videos, tracking logs for validating them by analyzing each vehicle and exporting trajectories, and extracted trajectory data. The dataset offers detailed trajectory data extracted from individual drone videos (54 datasets from nine flight campaigns, each involving six drones) and nine datasets of stitched footage combining data from all six drones. The data was extracted at a granularity of 30 frames per second. In addition to the trajectory data, the dataset includes the original video files and tracking logs, which provide visual context and enhance the usability and interpretability of the trajectory data. The tracking logs can be used to map vehicle IDs onto the video footage, facilitating various analyses as detailed in the accompanying user guide.

### Folder Structure

- **Data Folders:** Each flight campaign’s data is stored in a corresponding folder named “Data_T(flight campaign number), e.g., Data_T1, Data_T2. Within each folder:

– **CSV files** are named according to the drone in the format T(flight campaign number)_ D(drone number).csv, e.g., T1_D1.csv, T1_D2.csv.

– A file containing the stitched data from all six drones is named (e.g., T1_DAll.csv) for each flight campaign.

- **Tracking Logs and Videos Folders:** These follow a similar structure with folders for each flight campaign,e.g., Tracking_Logs_Videos_T1, Tracking_Logs_Videos_T2.

– **Video files** are named according to the drone and flight campaign, e.g., T1_D1.mp4, T1_D2.mp4

– **Tracking files** for individual drones are named, e.g., T1_D1.tlgx, T1_D2.tlgx

– The tracking file for the stitched footage from all six drones is named, e.g., T1_DAll.ftlgx

**Fig. 5:**
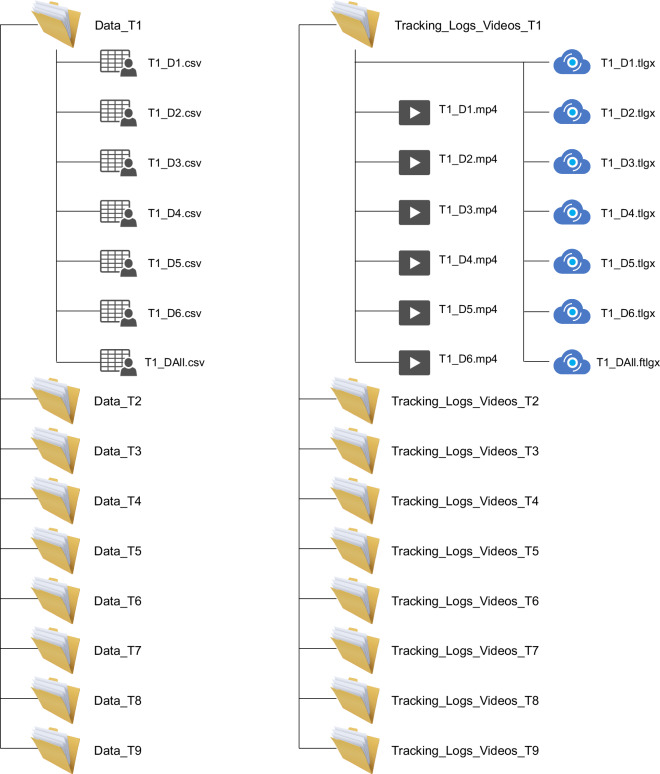
MiTra data repository folder structure.

The detailed folder structure is illustrated in Fig. 5.

To access the trajectory data, navigate to the “Data_T*” folder of the desired flight campaign, e.g., ("Data_T1”), to access the trajectory CSV files. Each CSV file contains trajectory data for individual vehicles, including timestamps, vehicle IDs, and position coordinates. In the trajectory data, in addition to standard variables like position, speed, and acceleration, the dataset includes a ‘Lane’ attribute for each vehicle at a given timestamp. This attribute identifies the lane occupied by the vehicle at each time step, covering: Mainline lanes: 0-3 for left-to-right traffic direction, and 4-7 for right-to-left traffic direction, Ramp lanes: 10-11 for inner and outer ramps (left-to-right), and 20-21 for ramps (right-to-left). This information lets users study lane-based traffic patterns, lane-changing behavior, and ramp interactions. To further enhance the dataset’s applicability, particularly for car-following and lane-changing studies, we have also added surrounding vehicle identifiers. For each vehicle, up to six IDs are provided: Leader_ID and Follower_ID (same lane), Left_Leader_ID and Left_Follower_ID (adjacent left lane), Right_Leader_ID and Right_Follower_ID (adjacent right lane). These are calculated based on the center point and heading direction of the subject vehicle. A value of -1 indicates the absence of a corresponding surrounding vehicle at that timestamp. Details of all variables are provided in Table 2.

**Table 2:** MiTra data attributes.

Column name	Description
Vehicle_ID	Unique vehicle ID for given flight campaign (and drone in case of single drone data)
Vehicle_type	Type of vehicle
Time [s]	Current timestamp
x [m]	UTM Coordinates longitude at the current time
y [m]	UTM Coordinates latitude at the current time
Speed [km/h]	Speed at the current time
Lon. Acc. [ms-2]	Longitudinal Acceleration at the current time in *m*/*s*^2^ (a positive value means acceleration and a negative value means deceleration)
Lat. Acc. [ms-2]	Lateral Acceleration at the current time in *m*/*s*^2^ (a positive value means acceleration to the right, and a negative value means acceleration to the left)
Angle [rad]	Heading angle
Vehicle_length [m]	Length of vehicle (Average for given type)
Vehicle_width [m]	Width of vehicle (Average for given type)
Lane	Lane number: 0-3: Mainline (left-to-right); 4-7: Mainline (right-to-left); 10-11: Inner/outer ramp (left-to-right); 20-21: Inner/outer ramp (right-to-left)
Leader_ID	ID of the leading vehicle in the same lane
Follower_ID	ID of the following vehicle in the same lane
Left_Leader_ID	ID of the leading vehicle in the left adjacent lane
Left_Follower_ID	ID of the following vehicle in the left adjacent lane
Right_Leader_ID	ID of the leading vehicle in the right adjacent lane
Right_Follower_ID	ID of the following vehicle in the right adjacent lane

## Technical Validation

The technical validation of our dataset was conducted through a series of rigorous analyses to ensure the accuracy, consistency, and reliability of the extracted vehicle trajectories. The validation process involved multiple approaches, each designed to assess a different aspect of the data processing pipeline.

### Validation of Vehicle Detection and Tracking

**Fig. 6:**
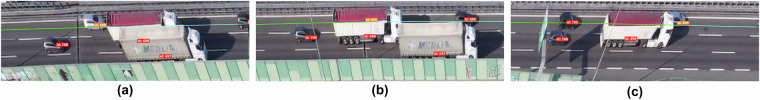
Screenshots from DataFromSky viewer showing accurate detection and tracking of occluded vehicles. (**a**) Vehicle 699 is visible before occlusion by Vehicle 698; (**b**) Vehicle 699 is fully occluded by Vehicle 698, yet accurately tracked; (**c**) Vehicle 699 reappears from occlusion with consistent tracking.

The DataFromSky team performed thorough manual cross-checking of the automated processes by comparing results with video ground truth. This manual intervention was essential in identifying and correcting any discrepancies, particularly in complex scenarios such as high traffic density or when vehicles or other objects occluded vehicles. For instance, Fig. 6 illustrates a scenario where a car (Vehicle 699 - Orange label) becomes occluded by a heavy vehicle (Vehicle 698- Red label) in an adjacent lane. As shown in Fig. 6(a), Vehicle 699 is visible and tracked before occlusion, as seen in the DataFromSky viewer. Figure 6(b) shows that Vehicle 699 is completely occluded by Vehicle 698, yet its position continues to be accurately tracked, and Fig. 6(c) shows Vehicle 699 reappears from behind Vehicle 698, with tracking accuracy maintained. These figures demonstrate the effectiveness of the combined automated and manual approach in maintaining accurate tracking even under challenging conditions.

**Fig. 7:**
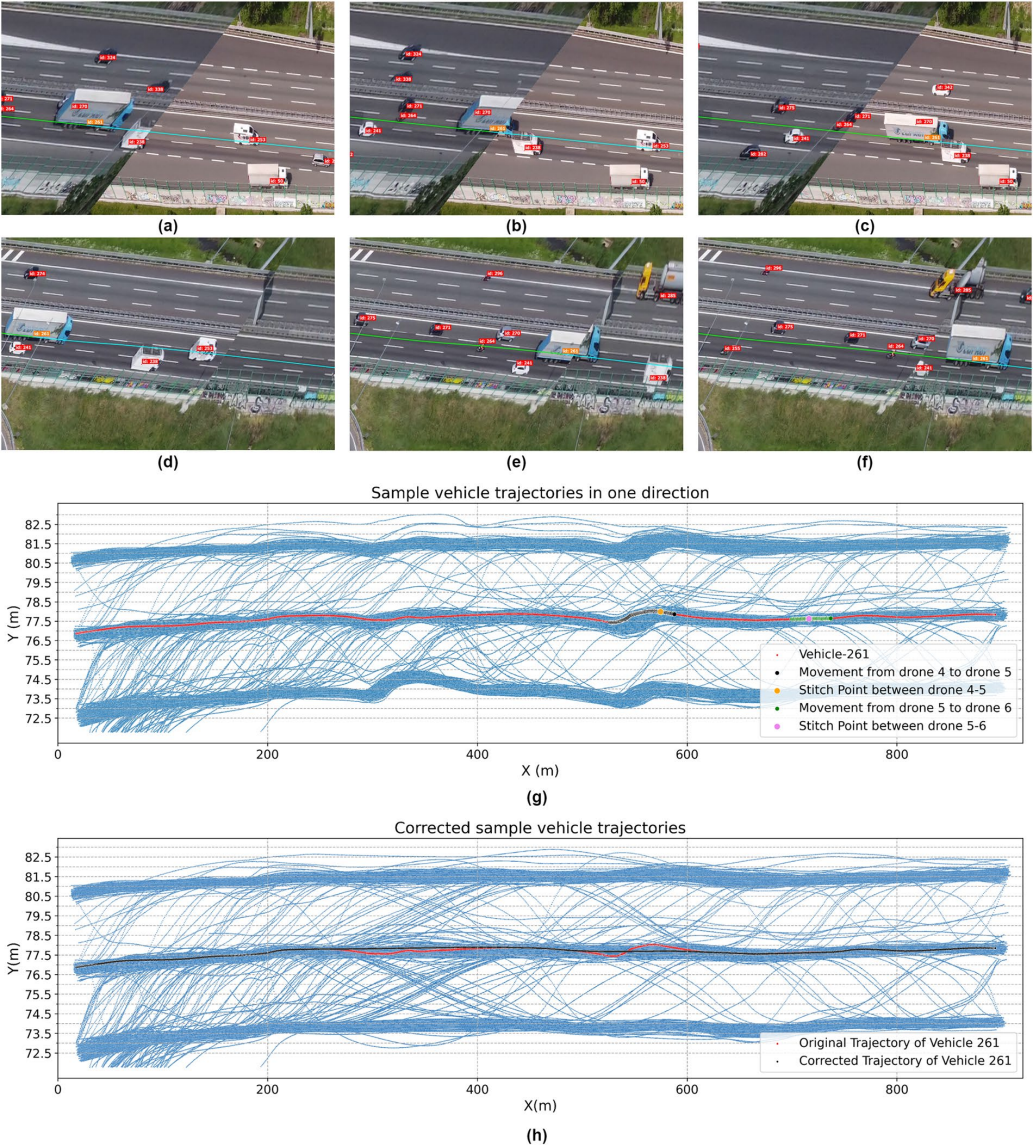
Validation of the stitching and trajectory extraction process. (**a**) Veh. 261 from Drone 5 point of view; (**b**) Veh. 261 at Stitch between Drone 5 and 6; (**c**) Veh. 261 from Drone 6 point of view; (**d**) Veh. 261 from Drone 4 point of view; (**e**) Veh. 261 at Stitch between Drone 4 and 5; (**f**) Veh. 261 from Drone 5 point of view; (**g**) Sample Trajectories; (**h**) Corrected Sample Trajectories.

The deep neural networks employed for vehicle detection were validated against a diverse dataset comprising real and synthetic images, ensuring high accuracy across various traffic conditions. Additionally, the MHT system, which maintained continuous trajectories, was rigorously tested to confirm its reliability, particularly in handling complex scenarios such as overlapping vehicles. A global positioning error of 25.3 cm is computed by using the average individual distance between 13 street lamp posts from the coordinates by Google Earth Pro (considered as ground truth) and the coordinates of the same lamp posts from the extracted data. The local error between vehicles is much smaller, as evidenced by Fig. 7(g).

### Validation of Video Stitching and Trajectory Extraction

The video stitching process was a critical step in ensuring the accuracy and continuity of vehicle trajectories as vehicles moved across different drone views. This process was validated through careful georegistration within the UTM coordinate system, which allowed for the alignment of video footage captured by adjacent drones. The georegistration ensured that vehicles were consistently re-identified as they transitioned from one drone’s field of view to another, thus maintaining continuous and accurate vehicle trajectories across the entire study section.

One of the key challenges in the video stitching process was ensuring the accuracy of vehicle identification and trajectory continuity across drones. Figures 7(a–c) illustrate this process using Vehicle 261(Orange label) as an example:

Figure 7(a) shows Vehicle 261 as observed from Drone 5’s perspective before reaching the stitch point between two adjacent drones. Figure 7(b) demonstrates the moment when Vehicle 261 crosses the stitch point between Drone 5 and Drone 6, with both drones capturing the vehicle simultaneously from different angles. Figure 7(c) captures Vehicle 261 after completely crossing the stitch point, now fully visible in Drone 6’s field of view. Notably, the vehicle’s identification and trajectory were maintained accurately throughout this transition. Further validation of the trajectory stitching is provided in Fig. 7(g), which displays the trajectories of all vehicles moving in one direction, with Vehicle 261 highlighted in red. The points where vehicles transition between Drone 5 and Drone 6 are marked in green, clearly showing that the trajectory of Vehicle 261, as well as other vehicles, was successfully extracted during the transition between these two drones. This demonstrates the effectiveness of the stitching process in maintaining trajectory integrity during drone transitions.

However, despite the accuracy of the stitching process in most cases, there were some challenges due to EASA regulations. These regulations prevented us from flying the drones directly above the study section, requiring us to fly them approximately 130 m to the side of the road. As a result, the drones captured the study section from different viewing angles, occasionally making it difficult to align the videos perfectly.

This issue is exemplified in Figs. 7(d–f), which also tracks Vehicle 261 but now focuses on the transition between Drone 4 and Drone 5:

- Figure 7(d) shows Vehicle 261 from Drone 4’s perspective, approaching the stitch point between Drone 4 and Drone 5.
- Figure 7(e) captures the vehicle as it crosses the stitch point between the two drones, and
- Figure 7(f) shows the vehicle in Drone 5’s field of view after completing the transition.

In this case, due to the difference in viewing angles and the difficulty in perfectly aligning the videos, a lateral shift of approximately 0.5 m was observed in the stitched video footage around 320 m and 580 m. This shift led to a slight jump in the extracted trajectory, as illustrated in Fig. 7(g) by the points marked in black. This lateral discontinuity can be observed for Vehicle 261, as well as other vehicles in the same section, especially when crossing between Drone 4 and Drone 5.

Although this lateral shift presents a challenge, it can be mitigated through global trajectory correction for all vehicles in the affected stretch. As shown in Fig. 7(g), the jump occurs consistently across vehicles in the three lanes, indicating that a systematic correction could be applied to adjust the lateral displacement and restore complete trajectory continuity across adjacent drone views. The corrected trajectories are shown in Fig. 7(h).

### Space-Time Plot Validation

**Fig. 8:**
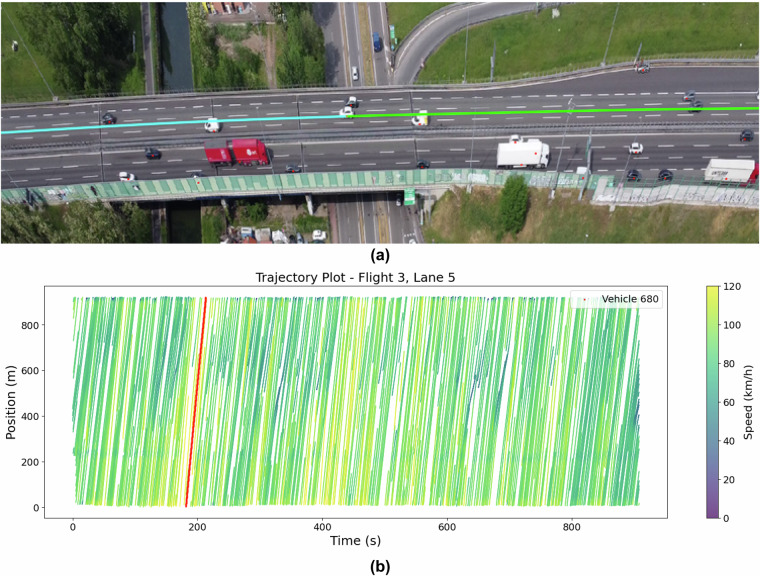
Observed traffic and corresponding trajectory plot for Flight 3. (**a**) Screenshot from Flight 3 video showing free-flow traffic; (**b**) Trajectory plot for Flight 3.

**Fig. 9:**
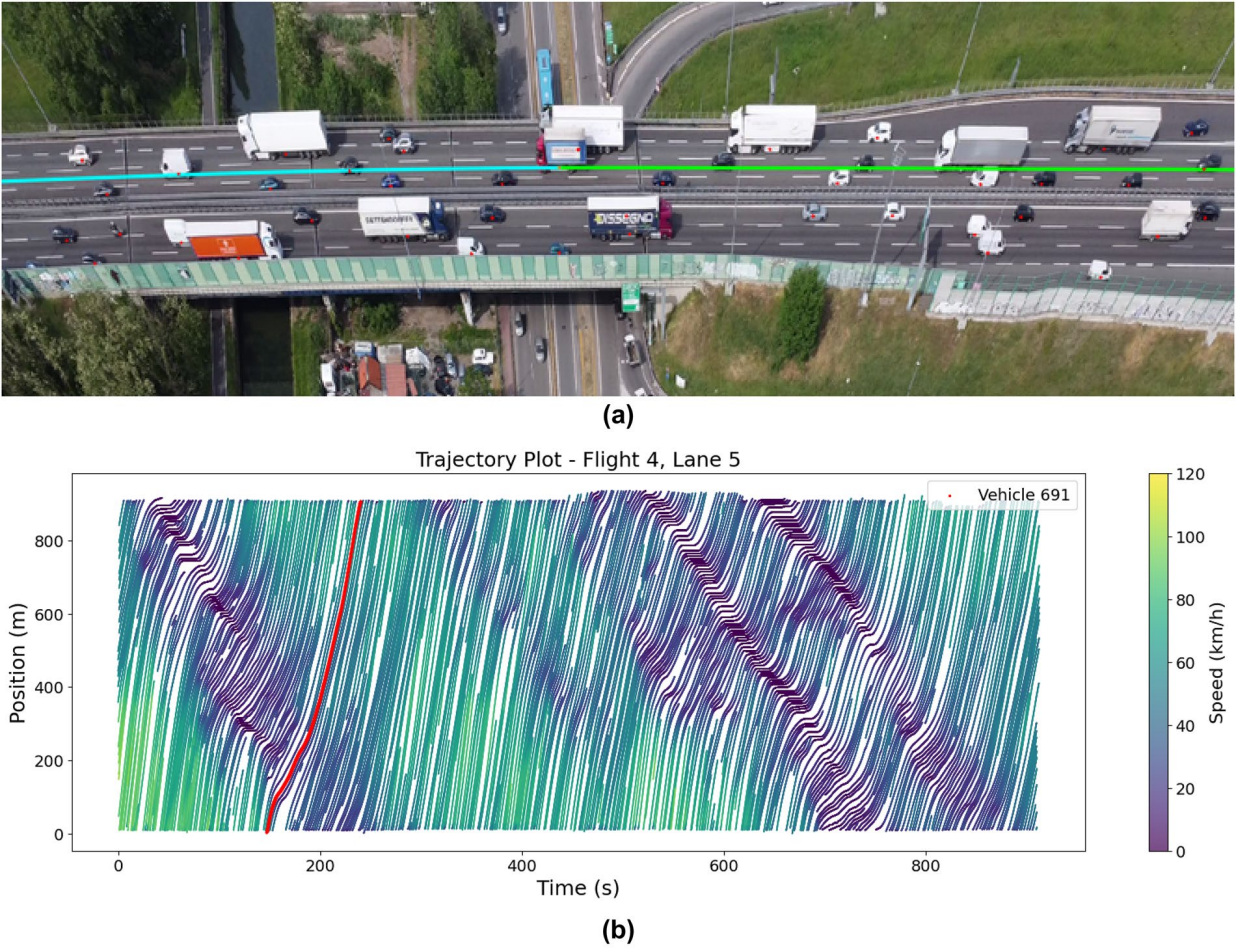
Observed traffic and corresponding trajectory plot for Flight 4. (**a**) Screenshot from Flight 4 video showing dense traffic; (**b**) Trajectory plot for Flight 4.

**Fig. 10:**
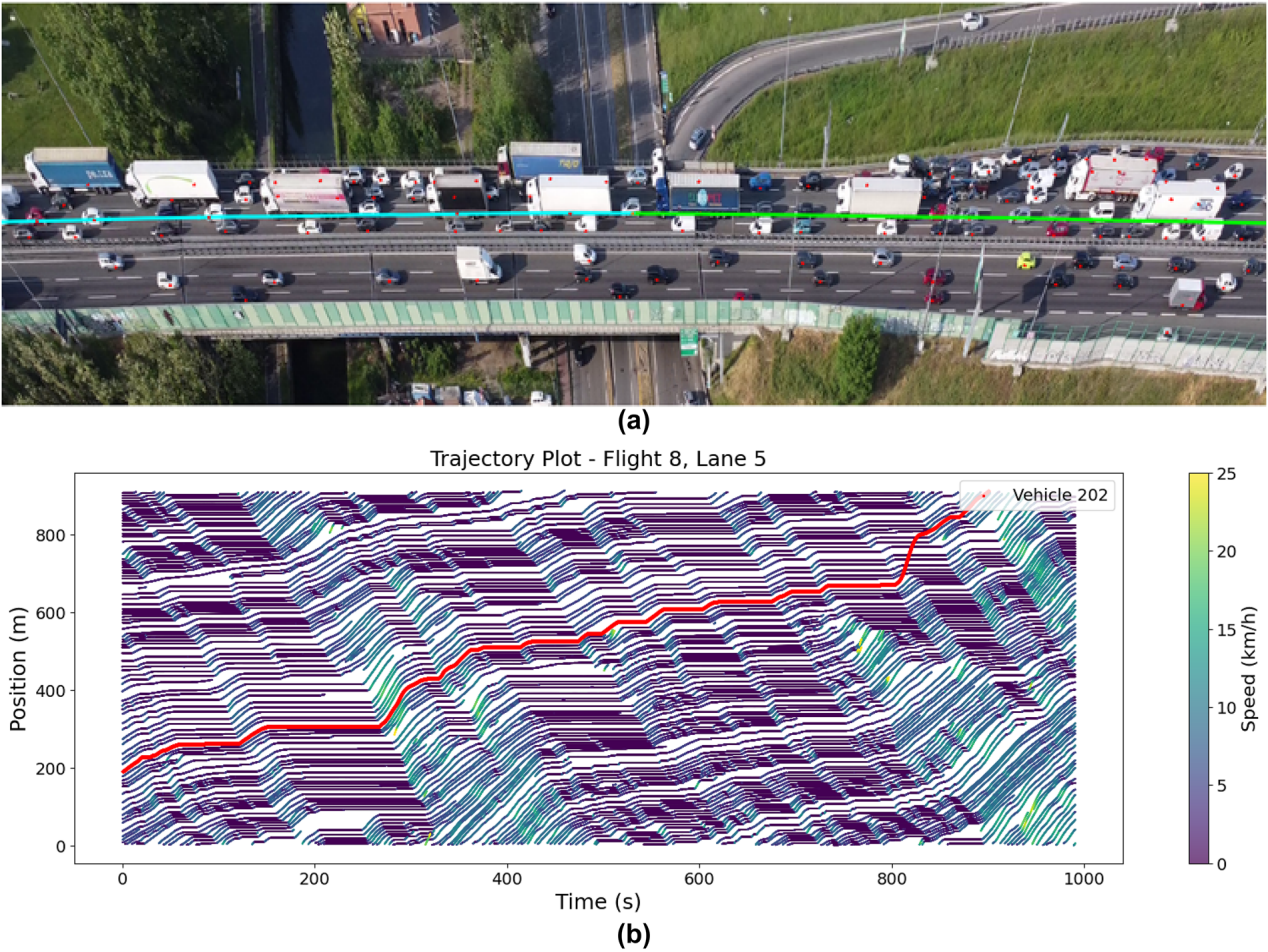
Observed traffic and corresponding trajectory plot for Flight 8. (**a**) Screenshot from Flight 8 video showing congested traffic; (**b**) Trajectory plot for Flight 8.

Space-time plots (or trajectory plots) were generated for different flights and compared with the corresponding traffic captured in the actual video footage taken simultaneously to validate the accuracy of the extracted trajectories further. Figures 8(a), 9(a), and 10(a) represent the screenshots of DFSViewer from the actual video for Flights 3, 4, and 8, respectively. The red dot on each vehicle is the identified tracked point whose location is recorded as the position of a vehicle; the green and blue (cyan) are completed, and future trajectories of a selected sample vehicle, respectively. Figures 8(b), 9(b), and 10(b) show the corresponding space-time plots for Flights 3, 4, and 8, respectively, with the red line showing the trajectory of the sample vehicle shown in Figs. 8(a)/9(a)/10(a).

The comparison between the plots and the videos revealed that the trajectories for free-flow conditions (Flight 3), dense traffic (Flight 4), and congested traffic (Flight 8) were accurately reflected in the space-time plots. The visual consistency between the video and the plots highlights the reliability of the dataset in capturing the various traffic states. For Flight 4, the space-time plot shows backward-propagating waves, with a wave speed of approximately 4.3 m/s. In Flight 8, where the traffic was fully congested with stop-and-go waves, the wave speed decreased to around 4 m/s, illustrating how wave speed reduces in more congested conditions. In both cases, the propagation waves in the space-time plots are parallel, showing uniform wave behavior in similar traffic conditions. The space-time plots provide a clear representation of traffic dynamics across different conditions, further validating the accuracy and consistency of the trajectory data. The close alignment between the visual evidence from the video footage and the generated plots ensures that this dataset reliably captures complex traffic scenarios, from free-flow to heavy congestion, across multiple lanes and traffic states.

### Speed-Flow-Density Analysis

We analyzed traffic dynamics using the classical relationships between speed, flow, and density to validate the dataset further, computed based on Edie’s method^37^. This approach accurately measures flow, speed, and density by averaging values across a defined space-time region. In this analysis, we considered a 100 m stretch along a single lane in the middle section (325-425 m) of the freeway, away from the influence of ramps, and used a 20 s time window for the calculations.

**Fig. 11:**
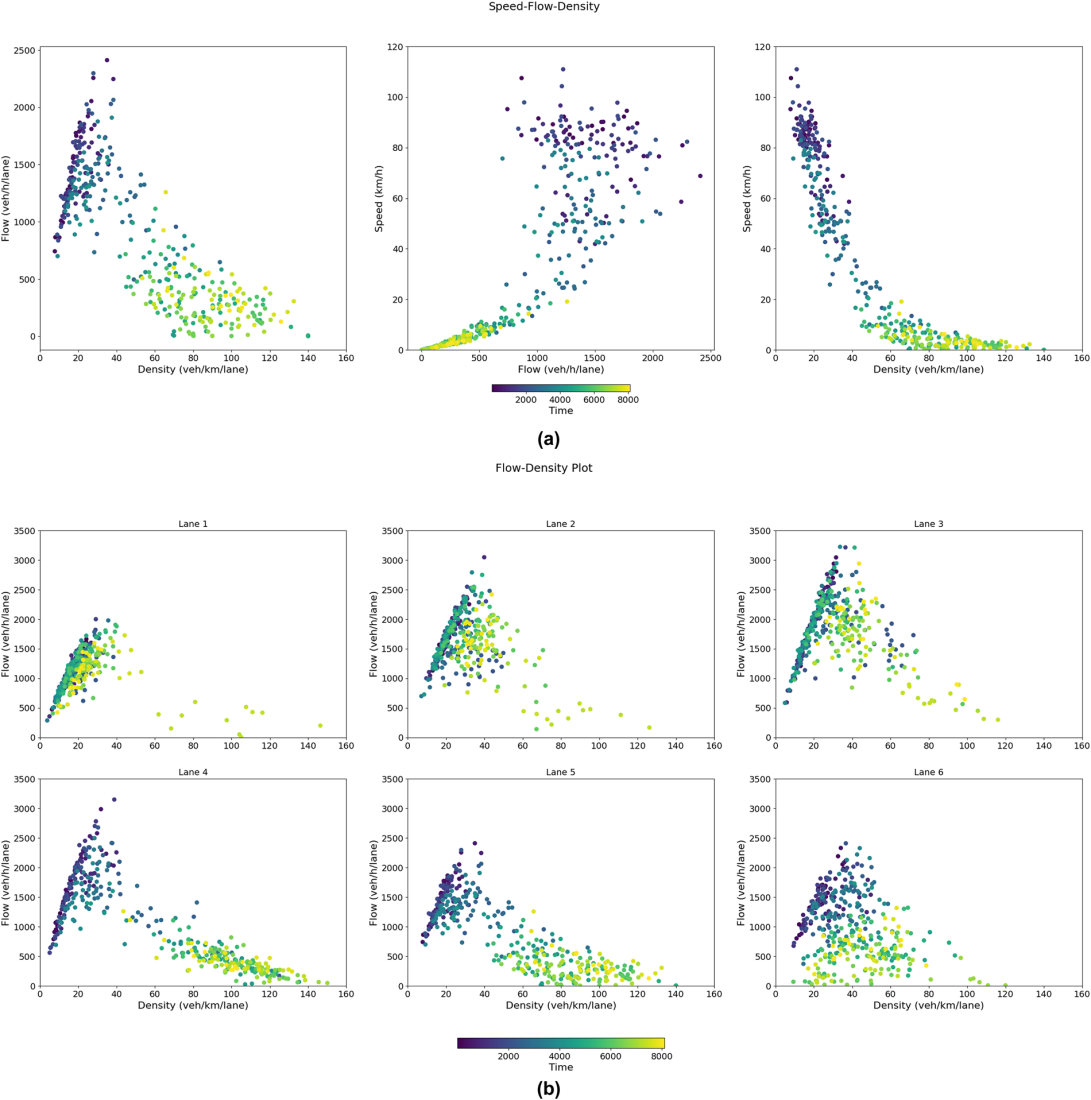
Speed Flow Density Distribution. (**a**) Density-flow, flow-speed, and density-speed scatter plots for lane 5 representing all traffic states. (These values are for all drones and all time slots (superset) data, a 100 m window is from 325-425 m of a stitched section, which is an exact 100 m section in the middle of Figs. 8-10.); (**b**) Density-flow scatter plot for different lanes.

Figure 11(a) presents scatterplots between these quantities for the selected section. The flow-density plot highlights both uncongested and congested traffic states. On the left side of the plot, the flow increases with increasing density, representing free-flow conditions. As density rises further, the flow reaches its peak before starting to decline, which marks the onset of congestion. This pattern reflects the typical behavior of traffic flow, where vehicles start interacting more as the density increases, eventually leading to a breakdown in flow. Additionally, from the flow-density plot in Figure 11(a), the wave speed of the traffic, indicating the speed of disturbance propagation through the congested traffic, is estimated to be around 4.6 m/s. Furthermore, from the speed-flow and speed-density plots, the relationship aligns with well-established traffic theory. The speed decreases as the density increases, particularly when traffic enters congested states. Similarly, high speeds are associated with lower flows in the speed-flow plot, while maximum flow occurs at an intermediate speed before declining in the congested regime. These plots confirm the transitions between different traffic states as captured in the dataset and show that the traffic behavior follows expected theoretical patterns. Notably, in Flight 8, as shown in the previous subsection, the trajectories displayed stop-and-go waves, indicating a highly congested state, which is further validated by the shape of these plots.

Figure 11(b) displays the density-flow plots for each major lane separately, providing additional insights into lane-specific traffic behavior. The plots reveal that lanes 1 to 3 (associated with the main traffic flow from left to right) mostly experience uncongested conditions, where flow increases steadily with density before tapering off. In contrast, lanes 4 to 6, which may be more heavily influenced by merging and diverging traffic, show congested conditions more frequently, with flow decreasing as density increases. These variations highlight the differences in traffic behavior between lanes, likely influenced by driver behavior, lane usage, and interactions with ramps.

**Fig. 12:**
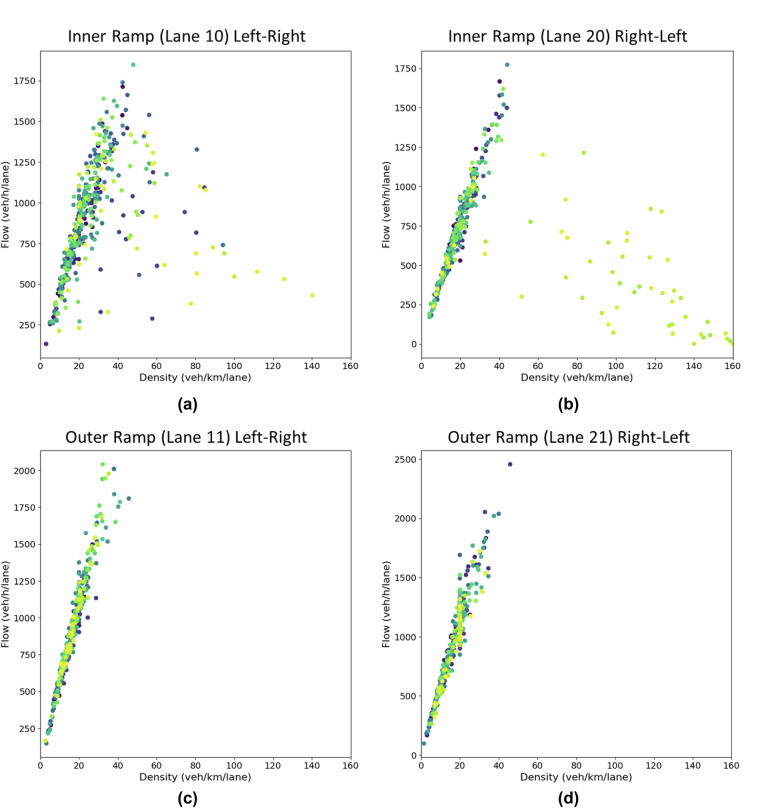
Density-Flow Distribution of Vehicles on Ramp. (**a**) Density-flow for inner ramp on left-right traffic; (**b**) Density-flow for inner ramp on right-left traffic; (**c**) Density-flow for outer ramp on left-right traffic; (**d**) Density-flow for outer ramp on right-left traffic.

Similarly, we have computed the density-flow plots for all four ramps—inner and outer ramps in both left-to-right and right-to-left directions—using Edie’s method on a 50 m section of each ramp. Figure 12 presents the density-flow distributions across these ramps. As shown in Fig. 12(a),(b), the inner ramps occasionally exhibit congested traffic conditions, particularly during the final flight campaigns. In contrast, Fig. 12(c),(d) illustrate that the outer ramps predominantly remain in the free-flow regime. These variations across different ramps demonstrate that the dataset successfully captures diverse traffic states, including merging, diverging, and queue-forming behavior, making it a valuable resource for detailed ramp-flow analysis.

The consistency of these speed-flow-density relationships provides strong validation for the dataset. The plots indicate that the traffic dynamics captured in the dataset reflect realistic traffic behavior, including transitions between free-flow, congested states, and stop-and-go conditions, further confirming the reliability of the data.

### Plausibility Checks and Data Integrity

The integrity of the data was further ensured through stringent plausibility checks. One such check was for vehicle teleportation, where the continuity of each trajectory was verified to prevent any sudden, unrealistic jumps in vehicle position with criteria of change in longitudinal position by more than 2 m in one-time step (i.e., a speed of 60 m/s) and lateral position by more than 0.5 m in one-time step (i.e., a speed of 15 m/s). In the case of single drone data, there are 26 instances of longitudinal jumps by three vehicles and 914 instances of lateral jumps shown by 595 vehicles out of 124 641 vehicles in 107 663 239 total timestamps. Similarly, for the stitched data cases, there are 27 instances of longitudinal jumps by three vehicles and 253 cases of lateral jumps by 60 vehicles out of 24 161 vehicles in 63 780 893 time instances. In summary, these checks of the data processing pipeline—from vehicle detection and tracking to video stitching and trajectory extraction—collectively demonstrate the robustness and reliability of the dataset.

## Usage Notes

For studies focusing on specific road segments or requiring high-resolution data, the single drone datasets offer a detailed view of vehicle trajectories within a narrower field. These datasets are particularly useful for analyzing micro-level interactions between vehicles. Conversely, the stitched data offers a broader view of vehicle movements across longer road stretches. It excels in extended microscopic analyses, particularly in complex scenarios like lane-changing, merging, and diverging. This data allows researchers to study how vehicles interact over longer distances, providing insights into the dynamics of traffic density, lane changes, and vehicle interactions in multi-lane environments. In summary, the trajectory data provided in this MiTra dataset is highly versatile and can be employed in various research areas, mainly:

- **Car-Following Models:** Due to its big size, diverse states, and high precision, the dataset is ideal for calibrating, validating, and improving car-following models, including the modeled anticipation and response to lane changes of others (passive lane changes).
- **Lane-Changing and Integrated Models:** The dataset includes both discretionary and mandatory lane changes, away from and near on-ramps and off-ramps, for all common traffic states. Particularly, it includes many merging and diverging events in congested situations, which is particularly challenging since effectively managing such situations implies awareness of and visual (or acoustic) communication and cooperation between all affected drivers, i.e., the lane-changing driver and at least the followers on the old and new lanes. Due to the high lateral precision, the data also provides an ideal testbed for integrated, fully two-dimensional microscopic models such as the Intelligent-Agent Model^38^. Specifically, the following aspects can be analyzed:

– Analyze how vehicles adjust their speeds and lanes when merging onto a highway or diverging onto an off-ramp.

– Develop and validate models that predict vehicle behavior during these scenarios, which are essential for improving the safety and efficiency of autonomous driving systems.

– Study the impact of merging and diverging traffic on overall traffic flow, particularly how these scenarios contribute to congestion and bottlenecks.

- **Algorithm Development for Autonomous Vehicles:** The dataset can be applied to the development and testing of algorithms for autonomous vehicles. This includes algorithms related to perception, prediction, and decision-making, especially in complex scenarios such as merging onto highways or navigating diverging lanes.
- **Urban Planning and Infrastructure Design:** The dataset can support urban planners and engineers in designing better road infrastructure by providing insights into how different road configurations (e.g., number of lanes, presence of ramps) affect traffic behavior. This is particularly relevant in evaluating the efficiency of current designs and proposing modifications to improve traffic flow at critical points like interchanges.
- **Safety Analysis:** Researchers can use the data to conduct safety analyses, focusing on high-risk scenarios such as merging and diverging. Due to the big data size, there are many instances of congested traffic, and with precision, researchers can identify rare events leading to near misses, associate these events with critical patterns, and develop strategies to mitigate these risks.

While the dataset provides a robust basis for analysis, users should be aware of important considerations of temporal discontinuity between flights. The data collected during the nine flight campaigns is not temporally connected. Each flight represents a separate and distinct dataset, with no continuous temporal link between the flights. As a result, the data from each flight should be analyzed independently rather than combined across flights. This is particularly important when considering longitudinal studies or analyses that require temporal continuity. Detailed information about each flight, including start and end times, can be found in Fig. 2(b).

## Data Availability

**Utilizing Tracking Logs with DataFromSky Viewer Software**. The tracking logs can be used in conjunction with the DataFromSky Viewer software, providing users with a powerful tool for visually inspecting the raw video footage while tracking individual vehicles. This allows for a detailed cross-checking of the trajectory data, enabling researchers to observe vehicle behavior in context, analyze surrounding traffic conditions, and interpret the reasons behind specific driving actions. The combined use of trajectory data and visual tracking provides a richer understanding of driver decision-making processes. Additionally, the software facilitates the extraction of key statistics, such as speed and acceleration, for further analysis. Accessing Tracking Logs: 1. **Install DataFromSky Viewer:** Before proceeding, ensure that you have installed the “DataFromSky Viewer” software. It can be downloaded from https://www.datafromsky.com/download/DataFromSkyViewer.exe, and the user manual (DFS_VIEWER_user_guide.pdf) is accessible through the software’s help section. 2. **Navigate to the Tracking Logs:** Access the desired flight campaign’s tracking logs by navigating to the “Tracking_Logs_Videos_T*” folder (e.g., “Tracking_Logs_Videos_T1”). 3. **Open the Tracking Log File:** Open the desired tracking log file. When prompted, select the corresponding video file with the same name as the tracking log (e.g., if you open the T1_D1.tlgx tracking log, choose the T1_D1.mp4 video file). 4. **Adjust Viewing Settings:** After loading the video in the DFS_Viewer software, adjust the settings for optimal viewing: Navigate to View  → Switch Coordinate Space  → Show in working coordinate space to obtain the best view of the tracked vehicles. 5. **Handling Stitched of Multiple Drone Tracking Files:** For tracking files involving all six drones (e.g., T*_DAll.ftlgx), select the video files in the following order for accurate synchronization: T*_D4.mp4, T*_D5.mp4, T*_D6.mp4, T*_D1.mp4, T*_D2.mp4, T*_D3.mp4. Follow the same viewing settings as described above to ensure the best results. **Working with the MiTra Dataset**. The Python script on https://github.com/ankitiitm/MiTra gives a sample of working with MiTra data for processing, visualization of the trajectories, and sample trajectory prediction using the IDM car-following model.
